# Lipid Rafts in Signalling, Diseases, and Infections: What Can Be Learned from Fluorescence Techniques?

**DOI:** 10.3390/membranes15010006

**Published:** 2025-01-01

**Authors:** Sara Anselmo, Elisa Bonaccorso, Chiara Gangemi, Giuseppe Sancataldo, Valeria Conti Nibali, Giovanna D’Angelo

**Affiliations:** 1 Department of Physics and Chemistry-Emilio Segré, University of Palermo, Viale delle Scienze, 90128 Palermo, Italy; sara.anselmo@unipa.it (S.A.); giuseppe.sancataldo@unipa.it (G.S.); 2 Department of Mathematics, Computer Science, Physics and Earth Science, University of Messina, Viale Stagno D’Alcontres 31, 98166 Messina, Italyvcontinibali@unime.it (V.C.N.)

**Keywords:** lipid rafts, liquid-ordered phase, fluorescent probes, super resolution, neurodegenerative disease, infections

## Abstract

Lipid rafts are dynamic microdomains in the membrane, rich in cholesterol and sphingolipids, that are critical for biological processes like cell signalling, membrane trafficking, and protein organization. Their essential role is claimed in both physiological and pathological conditions, including cancer, neurodegenerative diseases, and viral infections, making them a key area of research. Fluorescence-based approaches, including super-resolution fluorescence microscopy techniques, enable precise analysis of the organization, dynamics, and interactions of these microdomains, thanks also to the innovative design of appropriate fluorescent probes. Moreover, these non-invasive approaches allow for the study of live cells, facilitating the collection of quantitative data under physiologically relevant conditions. This review synthesizes the latest insights into the role of lipid rafts in biological and pathological processes and underscores how fluorescence techniques have advanced our understanding of these critical microdomains. The findings emphasize the pivotal role of lipid rafts in health and disease, providing a foundation for future research and potential therapeutic interventions.

## 1. Introduction

### 1.1. The Lipid Raft Hypothesis

The cell membrane was traditionally considered to be a homogeneous two-dimensional structure composed mainly of lipids and proteins [1]. According to this initial model, the thousands of lipid species present in the membrane were considered to have a purely passive and structural function, whereas membrane proteins were dynamic and played specific roles [2]. In 1997, Simons and Ikonen proposed the lipid rafts hypothesis, suggesting an active regulatory role for lipids in the plasma membrane. This hypothesis postulates that the distinct phase behaviours of various lipid species contribute to generating lateral heterogeneity in the lipid bilayer, facilitating the aggregation of specific proteins and modulating their diffusion [3].

The lipid rafts hypothesis describes the membrane as a system characterized by the coexistence of two liquid phases: a liquid-disordered phase (Ld), consisting mainly of unsaturated phospholipids, and a liquid-ordered phase (Lo), consisting of saturated phospholipids, sphingolipids, and cholesterol [4]. The interactions between saturated lipids, sphingolipids, and sterols in lipid rafts are stronger than in unsaturated lipids. Although these interactions are weak individually, their sum generates macroscopically relevant behaviour within the membrane [5].

The physicochemical basis of the lipid rafts hypothesis was developed through extensive studies of membrane models, which demonstrated how collective interactions between sterols and saturated lipids can create a unique state of matter characteristic of the Lo phase. This Lo phase is as fluid as the Ld phase, allowing some molecular mobility, but exhibits distinct physical properties, such as increased lipid packing, rigidity, and reduced permeability [6]. The Lo phase containing cholesterol allows for some lateral mobility of the lipids while maintaining a high degree of order. Cholesterol is, in fact, known to stabilize this phase by enhancing fluidity and maintaining an organized structure [7].

The lipid raft hypothesis has undergone modifications over time. In 2004, a model was proposed that emphasises small molecules and the nature of rafts. The lipid raft was considered a molecular complex of three molecules, including one with a saturated acyl chain or a cholesterol molecule with a critical role in the complex itself [8]. Recently, a definition was given that emphasised the dynamic nature of the lipid raft and the role of proteins (especially those of the cytoskeleton) in stabilising cell signalling processes. Several researchers have described lipid rafts as small (10–200 nm in size), dynamic, heterogeneous domains enriched in sterols and sphingolipids. Lipid rafts participate in cell signalling processes and, through protein–protein and protein–lipid interactions, form a larger platform [9]. In 2007, the field of research into these lipid domains was considered a ‘technical impasse’ because there were no physical instruments capable of studying lipid rafts [1].

In 2009, Davis et al. discovered, in addition to cholesterol, the presence of sphingomyelin (SM) in lipid rafts, as well as the interaction of cholesterol with ordered saturated lipids, including SM [10].

The lipid raft is no longer seen as a simple model based on a single physical process—phase separation—but as a set of elements that organize the complex structure of the membrane. It turns out that cholesterol is not the only element that defines the stability and organization of the membrane; there are other molecules, such as ceramide, that influence the order of the membrane [11].

Furthermore, over the years, gangliosides (GM), lipid-anchored proteins (such as GPI-anchored proteins), and transmembrane proteins have become essential components in the structure and function of lipid rafts. In particular, GM1, a specific class of glycosphingolipids, is involved in cell–cell communication and interacts with cholesterol-enriched domains in the plasma membrane. Moreover, GM1 has been shown, together with cholesterol, to play a role in initiating the aggregation of the amyloid peptide Aβ on the membrane [12].

Rafts are no longer seen as stable and well-defined micro-domains but rather as transient and dynamic regions that form only when necessary, such as during signal transduction or molecule transport. Lipid rafts can aggregate in response to specific signals and serve as platforms for the assembly of protein complexes, facilitating processes such as cellular communication and interaction with the external environment [13]. For example, they can promote the co-localisation of molecules such as enzymes and substrates or influence the conformation of proteins, modulating their activity [14].

### 1.2. Lipid Raft Functions

Lipid rafts organize membrane lipids and selectively cluster specific components, such as proteins, regulating their arrangement and interactions with other membrane constituents to facilitate various cellular processes [4]). 

**Figure 1:**
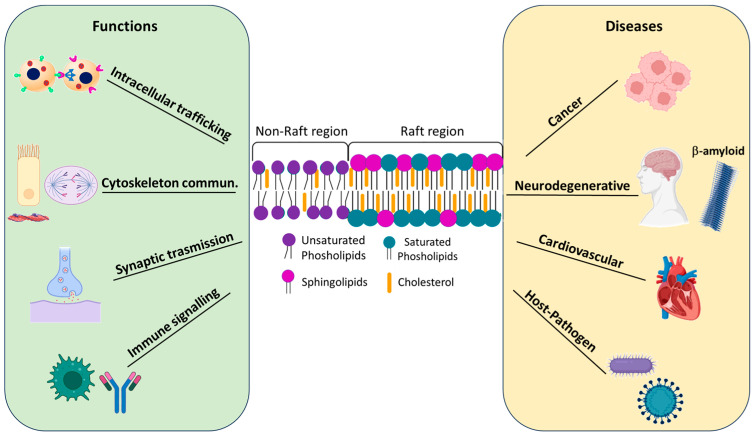
Schematic representation of lipid raft regions and their involvement in both cellular functions and diseases. Created with BioRender.com.

The main functions of lipid rafts are listed below, along with a schematic representation in Figure 1.

**Intracellular trafficking:** Lipid rafts play a crucial role in the classical endocytic pathway, particularly through a specific subgroup called caveolae. Caveolae are 50–100 nm diameter invaginations formed by specific caveolin proteins whose oligomerization causes membrane invagination, facilitating the internalization of membrane components and extracellular ligands [15]. Recent studies have demonstrated the involvement of raft domains in the sorting of molecules into exosomes (EXO), small extracellular vesicles that transport molecules between cells and tissues. Exosomes can facilitate the intercellular transfer of toxic proteins, such as Aβ peptide, tau, and alpha-synuclein, involved in the pathogenesis of Alzheimer’s, frontotemporal dementia, and Lewy body dementia [16]. Several researchers have observed the influence of environmental toxicants on signalling mechanisms associated with lipid rafts. Their data suggest that raft domains may serve as a platform for the regulation of damaging cellular mechanisms activated by these chemicals [17].

**Communication with the cytoskeleton:** Interactions between lipid rafts and the cytoskeleton are crucial for various cellular functions, such as signalling, migration, and cell adhesion [18]. Various microtubule stabilizers, such as Rho, RacGTPase, cadherin, actin, tubulin, and myosin, have been isolated in lipid rafts domains [19]. A relevant example is viral infection, during which lipid rafts interact with the PI3K signalling pathway to facilitate virus entry. Viruses bind to receptors associated with lipid rafts, activate the PI3K pathway, and promote actin polymerisation and rearrangement, facilitating virus endocytosis. Numerous antivirals target this interaction to limit viral infections [20].

**Synaptic transmission:** Lipid rafts are present in neurons and glial cells, where they participate in neuronal communication. They contribute to the exocytosis of neurotransmitters in presynaptic terminals and to the formation of neurotransmitter receptor clusters and their downstream effectors. Neurotransmitter release is influenced by cholesterol levels, with a correlation between cholesterol and synaptic plasticity mediated by proteins present in both raft and non-raft domains [21].

**Immune signalling:** Lipid rafts modulate both innate and adaptive immune responses. Receptor proteins of B and T lymphocytes, basophils, and mast cells are located within lipid rafts, where they are involved in signalling pathways leading to inflammation. Several GPI-anchored proteins associated with the immune system prefer to interact with lipid rafts. Furthermore, lipid rafts influence cytokine signalling by compartmentalising their receptors, creating signalling platforms that facilitate the recruitment of kinases and adaptor molecules to regulate cytokine activity [22].

### 1.3. Lipid Rafts and Diseases

Numerous researchers have shown that lipid rafts are related to the onset of various diseases, such as those reported below and in Figure 1.

**Cancer:** Cancer cells have numerous lipid rafts that are rich in cholesterol. The presence of lipid rafts in cancer cells is important because lipid rafts can serve as platforms for ordering various signalling processes and concentrate many cancer-related signalling and adhesion molecules. Lipid rafts are also involved in important processes related to cancer progression and therapy [23]. Lipid rafts contribute significantly to the adhesion and migration processes of tumour cells. The alternation between cell adhesion and migration is closely linked to aggressive invasion and metastatic spread of the tumour, phenomena that involve complex remodelling of the extracellular matrix of the tumour microenvironment and the involvement of adhesion molecules present on the surface of cancer cells [24]. Numerous studies have shown that CD44, the main cell adhesion protein expressed by cancer cells, is localised in lipid rafts and that the functionality of this protein is regulated by the presence of these lipid structures [25].

Lipid rafts organize integrins and influence their activity. Integrins interact with proteins involved in the cytoskeleton, such as Src family kinases (SFK). These kinases are activated within the lipid rafts, facilitating signal transduction downstream of the integrins [26].

CD95 and tumour necrosis factor (TNF) receptors are associated molecules in lipid rafts and are involved in apoptosis, a programmed cell death that is essential for the development and prevention of cancer.

Therefore, lipid rafts, through these processes, can interfere with the development of cancer and metastasis [27]. Several researchers have demonstrated the existence of potential prognostic and diagnostic markers in lipid rafts, from the epidermal growth factor pathway in HER2+ tumours to hormone receptor-positive subtypes that could be useful for the diagnosis and treatment of breast cancer [28].

**Neurodegenerative diseases:** Molecular alterations in lipid rafts are associated with neurodegenerative diseases such as Parkinson’s disease (PD), Alzheimer’s disease (AD), Prion diseases, and amyotrophic lateral sclerosis. These modifications of lipid rafts trigger processes involved in amyloid genesis, aberrant protein misfolding, and toxicity signalling, particularly in the early stages of several diseases. Several disease-triggering proteins, such as alpha-synuclein (αS) for PD, Aβ (amyloid-beta-peptide) for AD, and the prion protein PrPc, are integrated into lipid rafts [29]. In AD, the amyloid precursor protein (APP), β-(BACE), and γ-secretase are involved in the production and aggregation of the Aβ peptide. APP moves to lipid rafts, where it associates with flotillin 1, thus influencing amyloid generation. In addition, BACE and γ-secretase regulate the release of Aβ by interacting with lipids in the lipid raft [30].

**Cardiovascular diseases**: Lipid rafts are involved in heart disease, affecting receptor signalling in endothelial cells of the arteries and heart muscle. Lipid rafts play an important role in the pathogenesis of atherosclerosis, a vascular disease characterized by excessive cholesterol deposition in the artery wall and uptake by macrophages. Cholesterol uptake by immune cells is mediated by lipoproteins; the LDL receptor, CD36, is located in lipid rafts [31].

**Host–pathogen interactions**: During the infection process, pathogens use cellular lipids to complete their life cycles. They bind to the host’s plasma membrane and release infectious particles inside the cells. Both pathogenic microorganisms and viruses, bound and unbound, utilise the cholesterol-rich domains of lipid rafts to facilitate entry into the host cell and as platforms for the assembly of the viral envelope. This mechanism occurs due to the high concentration of cellular receptors on lipid rafts, which become targets for bacterial and viral molecules, such as cholera toxin, allowing pathogen penetration and strategic positioning of viral envelope components during assembly [32]. A correlation between lipid rafts and SARS-CoV-2 pathogenesis has recently been discovered. Infection causes metabolic remodelling of host cell lipids. Lipidome changes in infected patients and their relevance as potential diagnostic or prognostic clinical biomarkers have been investigated [33]. It was shown that the depletion of membrane-bound cholesterol from the lipid raft of ACE2-rich cells (the receptor where the virus binds) impaired the entry of SARS-CoV-2 into host cells [34].

Section 6 will report some case studies demonstrating the potential of fluorescence techniques in highlighting the role and properties of lipid rafts in different human diseases.

## 2. Methods of Studying Lipid Organization

Over the course of the last few years, the application of a plethora of experimental techniques led to a renewal of the lipid raft hypothesis. Raft-like domains have been investigated through sub-micrometre-scale-sensitive techniques, including neutron scattering [35] and nuclear magnetic resonance [36,37,38]. Studies by atomic force microscopy (AFM) have allowed the comprehension of certain properties of the nanodomains [39,40]. For example, Drolle et al. combined AFM with Kelvin probe force microscopy (KPFM) to investigate the complex model of lipid membranes that mimic healthy, early- and late-stage AD using 5-component mixtures of 1,2-dipalmitoyl-sn-glycero-3-phosphocholine (DPPC)/ 1-palmitoyl-2-oleoyl-sn-glycero-3-phosphocholine (POPC)/CHOL/SM/GM1 in different ratios [41]. Furthermore, calorimetric techniques, such as differential scanning calorimetry, have provided fundamental data that, on the one hand, have confirmed the existence of membrane domains [42] and, on the other hand, combined with other techniques, have enabled the construction of phase diagrams of different lipid mixtures [43].

**Figure 2:**
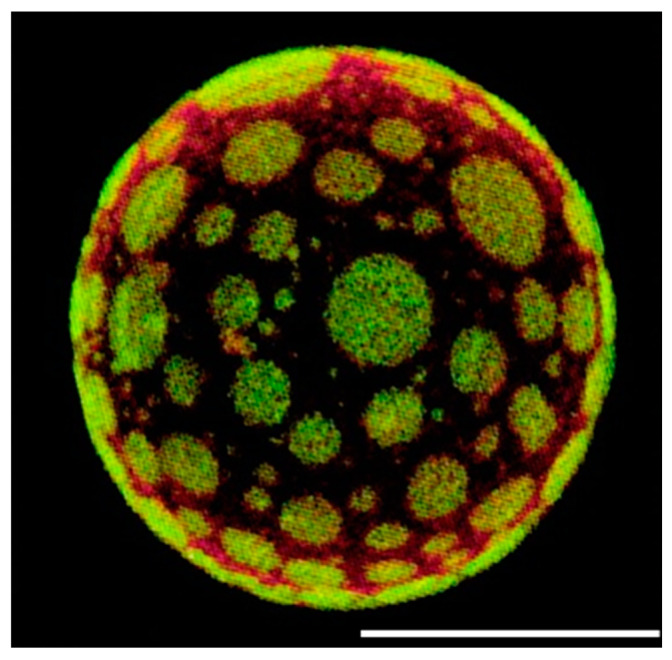
Representative confocal fluorescence image of giant unilamellar vesicle formed from native pulmonary surfactant membranes, labelled with the lipophilic fluorescent probes DiIC18 (red) and Bodipy-PC (green). Adapted from Ref. [44] 2004. The green areas correspond to the liquid-disordered regions and the red background corresponds to the liquid-ordered phase. The scale bar corresponds to 20 μm.

While a variety of techniques can be used to study membrane phase behaviour, optical microscopy remains the gold standard for in situ studies of lipid rafts. A representative image is reported in Figure 2.

Notably, fluorescence microscopy has emerged as a powerful tool for visualizing these dynamic membrane domains.

Fluorescence is the emission of light that occurs within nanoseconds after the absorption of light that is typically of a shorter wavelength [45]. After the 1960s, great technological advances and methods based on the phenomenon of fluorescence were made, such as confocal microscopy, fluorescence recovery after photobleaching (FRAP), Forster Resonance Energy Transfer (FRET), and two-photon microscopy, that not only revolutionized imaging but also yielded access to dynamics on previously inaccessible length- and time-scales [46]. Thanks to the dramatic improvements in sensitivity and resolution, fluorescence techniques have significantly advanced our understanding of lipid raft dynamics and function.

Since biological systems are poor in intrinsic fluorescent species, optical microscopy requires the use of contrast agents and fluorescent probes [47]. The need for fluorescent labelling remains a limitation since it can potentially perturb the native state of the membrane. For this reason, the reliance on fluorescent probes introduces the challenge of developing suitable fluorophores that do not affect the system’s behaviour.

## 3. Fluorescent Probes for Studying Lipid Rafts

**Figure 3:**
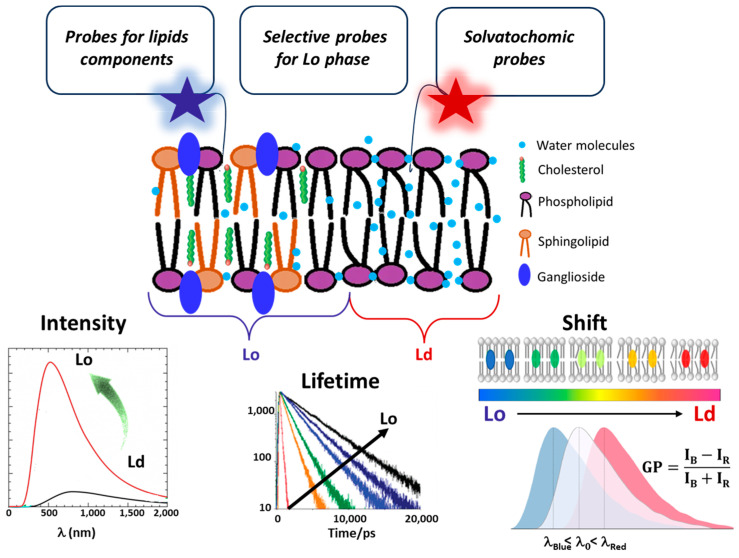
Schematic representation of the lipid bilayer, illustrating the distinction between the liquid-disordered (Ld) and liquid-ordered (Lo) phases. The Lo phase is enriched in cholesterol, sphingolipids, and gangliosides, which are key components of lipid rafts. Responses of fluorescent probes: the spectral parameters change depending on the stiffness, polarity, and viscosity of the membrane.

Fluorescent probes are powerful and widespread tools in biophysics that are widely employed for functional imaging in vitro and in vivo. Here, we are interested in their use for probing lipid rafts. Fluorescent probes fall into three primary categories, each tailored for specific experimental applications [47,48]. The first includes probes that bind specifically to lipid molecules, allowing for their direct visualization within the membrane. The second category contains probes that selectively localize within Lo or Ld lipid phases, aiding in mapping structural organization. The third group comprises environment-sensitive probes, including solvatochromic dyes and molecular rotors, which can distinguish Lo and Ld phases by responding to local fluidity, polarity, and viscosity. However, these parameters are interconnected, as changes in membrane fluidity often affect both polarity and viscosity and vice versa [49,50,51]. This interconnection can complicate the interpretation of results, requiring careful consideration of how these factors interact in the context of membrane dynamics and organization. Table 1 provides a summary of the main fluorescent dyes, categorized into three distinct classes. Meanwhile, Figure 3 provides an overview of the spectral properties of the probes, which are monitored to differentiate between the Lo and Ld regions.

### 3.1. Probes Selective for Lipid Components

Lipid rafts in cell membranes, as discussed above, are enriched in GM1 gangliosides, SM, and cholesterol, which confer distinct structural and functional properties to these microdomains. The use of probes selective for these components allows for direct visualization and in-depth analysis of these regions. One of the most established and widely used probes in this category is the fluorescently labelled Cholera toxin B (CT-B), which binds to GM1 gangliosides [52], facilitating targeted observation of these ordered membrane regions. Beyond localization, CT-B has been instrumental in studying protein–lipid interactions and their effects on membrane structure and function. Norbert Blank and colleagues demonstrated CT-B’s ability to modulate membrane dynamics by reorganizing lipid rafts, influencing critical cellular functions such as signal transduction and membrane trafficking, involved in both normal physiology and various disease states [81]. However, while CT-B is widely used, it presents limited specificity, as it can also bind to other sugar moieties on the cell surface, like galactose and lactose [82]. An alternative to CT-B is BODIPY-GM1, a fluorescently modified version of GM1 that incorporates the BODIPY dye at the N-acyl chain position of the ceramide. The fluorescent BODIPY core is chemically robust, allowing extensive modifications to the meso-phenyl ring without degrading the dye [83]. BODIPY-GM1 retains the natural targeting specificity of GM1 gangliosides, integrating seamlessly into cell membranes. This quality makes it highly suitable for real-time observation of GM1 distribution and lipid raft behaviour in live cells without compromising membrane integrity. For instance, Ilya Mikhalyov and Andrey Samsonov used BODIPY-GM1 to analyse lipid raft dynamics in living red blood cell membranes under fluorescence microscopy, providing critical insights into membrane organization and stability [54]. 

To visualize cellular cholesterol organization and to measure dynamics (such as uptake, diffusion, trafficking, and localization) with high specificity and sensitivity, fluorescent cholesterol-binding molecules, including filipin [56], intrinsically fluorescent mimics of cholesterol, such as cholestatrienol (CTL) [58] and dehydroergosterol (DHE) [60,84], or organic dye-labelled cholesterol analogues are used [59]. Filipin, a polyene antibiotic, binds to cholesterol molecules [56,57] and fluoresces upon interaction, making it a standard tool for mapping cholesterol distribution in cell membranes and investigating cholesterol disorders [57]. Bruno et al. utilized filipin as a diagnostic marker to investigate cholesterol dysregulation in disorders such as Niemann–Pick Type C disease and several neurodegenerative conditions [85]. However, filipin also has limitations: Its cholesterol binding is not entirely specific, as it may interact with other lipids or membrane proteins [55], leading to potential misinterpretations. Additionally, at high concentrations, filipin can disrupt membrane integrity [86], which complicates its use in live-cell studies. Thus, careful optimization is required for reliable visualization, limiting its use across diverse experimental conditions.

Natural cholesterol analogues such as DHE and CTL are, in principle, the most suitable molecules for mimicking cholesterol [58,59,84]. They can be inserted specifically into the plasma membrane or delivered to cells as part of lipoproteins for subsequent analysis of their transport itineraries and metabolism [59]. However, their photophysical properties, including UV excitation, low quantum yield, and high photobleaching, make them challenging for prolonged microscopy or single-molecule fluorescence fluctuation studies [59]. Organic dye-labelled cholesterol analogues, like 7-nitro-2,1,3-benzoxadiazol-4-yl (NBD)-cholesterol [61] and TopFluor-Cholesterol (TF-Chol), a BODIPY-labelled cholesterol analogue [62,87], offer higher fluorescence and are favoured for their ability to mimic cholesterol’s distribution in membranes.

To study SM, fluorescent SM analogues, designed to closely mimic the behaviour of natural SM, are employed. Notable examples include BODIPY-labelled SM [63] as well as ATTO_488_ and ATTO_594_-labelled SM probes [64]. Masanao Kinoshita and colleagues demonstrated that the combination of ATTO_488_-SM and ATTO_594_-SM allows, via FRET analysis, clear visualization of SM-rich domains in both synthetic lipid vesicles and live cell membranes [64]. This approach significantly advances our understanding of SM distribution and dynamics in cellular environments.

### 3.2. Probes Selective for the Lo Membrane Phase

Since the aim of this review is to analyse lipid rafts, we will focus on dyes selective for the Lo phase. By selecting probes that preferentially associate with the Lo phase, we can better visualize and study these membrane domains and their roles in cellular processes.

The probes used can be categorized into two main types. The first category includes fluorescently labelled lipids, such as BODIPY-SM and TF-Cholesterol, mentioned above, which preferentially associate with the Lo phase. In contrast, the other dyes, like ATTO-SM and NBD-cholesterol, which contain more polar ATTO and NBD groups compared to BODIPY, tend to partition into Ld phases [65]. This makes them less suitable for targeting lipid rafts directly.

The second family corresponds to lipophilic fluorescent molecules of a nonlipid nature, primarily represented by fluorescent polycyclic aromatic hydrocarbons (PAHs) [65]. The most well-known PAH dyes, including terrylene and naphthopyrene (NAP), possess a planar molecular structure that enables them to efficiently intercalate between lipids within the Lo phase. This behaviour is analogous to cholesterol and results in a distinct preference for these ordered domains [65]. Tobias Baumgart et al. demonstrated that NAP exhibits stronger partitioning into Lo phases within the brain sphingomyelin system [65]. Additionally, Janos Juhasz and colleagues used this dye to investigate the structure and dynamics of lipid rafts in synthetic membranes, particularly in cholesterol-rich giant unilamellar vesicles [88].

### 3.3. Environment-Sensitive Dyes

Environment-sensitive dyes change their spectroscopic properties in response to environmental parameters, such as polarity, hydration, viscosity, etc. [89]. In this context, two classes of probes should be mentioned: solvatochromic probes and viscosity-sensitive probes (molecular rotors).

The former change their colour in response to the polarity of the environment, while the latter probes change their fluorescence intensity and lifetime in response to environmental viscosity [47]. Because the Lo phase is characterized by a higher level of lipid packing compared to the Ld phase, it is also much less hydrated (i.e., less polar) and more viscous. Therefore, both solvatochromic and viscosity probes can distinguish Lo from Ld phases.

***Solvatochromic dyes*** are powerful tools for studying lipid rafts due to their sensitivity to the surrounding environment’s polarity, allowing them to detect subtle variations in membrane organization and lipid composition. These dyes change their fluorescence emission based on the local lipid environment, making them particularly useful in distinguishing between ordered and more disordered areas of the membrane.

Dyes like Laurdan (6-lauroyl-2-dimethylaminonaphthalene) and Prodan (6-propionyl-2-dimethylaminonaphthalene) are widely chosen for this purpose [68]. Their spectral properties change in response to lipid packing, and a blue shift of around 50 nm in the emission peak for both dyes is observed for membranes in the Lo phase (few water molecules in the bilayer) relative to membranes in the Ld phase (more water molecules in the bilayer) [68,69,90]. Indeed, when the dyes are in membranes characterized by the Ld phase, the energy required for solvent reorientation is higher and decreases the excited state energy of the probes [90]. This is reflected in a redshift of the probe’s emission spectrum, which can be quantified by the Generalized Polarization (GP) function [49,67,91] defined as

$$GP=\frac{I_{B}-I_{R}}{I_{B}+I_{R}}$$

where *I_B_* and *I_R_* are the emission intensities at the blue and red edges of the Laurdan emission spectrum, respectively. *GP*, therefore, defines a parameter constituted by a ratio of the two emission intensities (at the blue and green edges of the Laurdan emission spectrum) for a given excitation wavelength. GP values range from −1 to +1, indicating low to high membrane order [49,66,92]; these values can shift in response to variations in membrane fluidity due to molecules interaction with the bilayer [49,93,94,95]. However, these dyes are not selective for a specific leaflet of the membrane. Both are neutral hydrophobic molecules that can migrate, or “flip-flop”, across the bilayer, meaning they are able to distribute between both the outer and inner leaflets [89].This lack of leaflet specificity makes them suitable for studying overall membrane order and phase properties but not for experiments requiring selective targeting of just one leaflet [47]. In addition, they were found to behave ideally in model membranes but not in cells since data analysis is complicated by the rapid internalization of this dye [47].

To enhance membrane studies by increasing probe lipophilicity and stability within the membranes, an extended aliphatic chain was added to Laurdan, producing a more hydrophobic derivative, C-Laurdan, better suited for deeper integration within the lipid bilayer [70,96]. The longer hydrophobic tail allows C-Laurdan to embed more stably within the membrane, significantly reducing its tendency to diffuse into the surrounding aqueous environment. Additionally, C-Laurdan demonstrates greater sensitivity to solvent polarity and exhibits stronger fluorescence emission compared to Laurdan [70].

All dyes in this class discussed thus far are excited by near-ultraviolet light, making them ideal for multiphoton microscopy at around 800 nm. This approach offers a significant advantage in cellular applications by limiting photobleaching and minimizing the damaging effects typically associated with UV excitation [94,97]. Only dye molecules within the optical section are excited, preserving signal quality and reducing exposure outside the focal plane. However, multiphoton excitation requires complex and costly equipment, which can be a limitation.

To overcome this, Di-4-ANEPPDHQ provides a convenient alternative. It can be excited by blue light, including the commonly available 488 nm laser line used in most confocal microscopes [97]. Moreover, di-4-ANEPPDHQ has two positive charges on its headgroup, which retard the rate of flipping from the outer leaflet of the bilayer to the inner leaflet [71]. This overcomes the problems discussed above and is associated with many membrane probes that work perfectly in model membranes but cannot really be applied to cell membranes because of their rapid intracellular entry, their flip-flopping between the two leaflets, and their mispartitioning between Lo and Ld phases [47]. Although Di-4-ANEPPDHQ operates by the same mechanism as Laurdan [49,50,98], these two dyes have been observed to report at different depths in the phospholipid model bilayers [49,99,100]: the fluorescent naphthalene moiety of Laurdan probes the interphase region between the lipid head groups and the first C-atoms of the hydrophobic acyl chains, whereas the chromophore of di-4-ANEPPDHQ aligns with the acyl groups deeper in the hydrophobic core. Based on this, Laurdan and di-4-ANEPPDHQ were used together by Anselmo and coworkers to analyse, at different depths, the effect of the TP10 peptide on membrane order [49]. Another notable dye in this family is Nile Red (NR), an uncharged and hydrophobic fluorescent dye whose fluorescence properties change depending on the dielectric constant of its environment and are studied to analyse how membrane order and fluidity are affected by lipid composition, chain saturation, and the presence of cholesterol [72,101,102]. NR absorption, fluorescence lifetime, and quantum yield are sensitive to solvent polarity, which correlates with changes in membrane fluidity [50]. As membrane fluidity increases, a greater number of water molecules penetrate the lipid bilayer, exposing NR to a more hydrophilic environment [50]. However, NR exhibits less specificity for lipid rafts, exhibiting a broader binding profile, and is unsuitable for studying cell plasma membranes because it readily internalizes into cells and specifically binds to lipid droplets, the most apolar compartments within cells. To achieve selective localization of the fluorophore at the plasma membrane, NR can be conjugated with a zwitterionic group and a long hydrophobic chain.

Oleksandr A. Kucherak and coworkers modified the NR by adding an amphiphilic anchor group, creating the new probe NR12S [73]. They demonstrated that NR12S exclusively stains the outer leaflet of lipid vesicles, with minimal flip-flop across the bilayer. The probe in model vesicles exhibits distinct emission spectra in Lo and Ld phases, making it effective for distinguishing these membrane regions, and in living cells, its emission colour varies with the cholesterol content; thus, this probe constitutes a new tool for quantification of lipid rafts in cell plasma membranes. Another dye that localizes to the outer membrane leaflet is F2N12S, which, similar to NR12S, has a long hydrocarbon chain and a zwitterionic group [74]. In the study by Andrey S. Klymchenko et al., the probe F2N12S demonstrated the ability to partition into both Lo and Ld phases of giant vesicles. This partitioning allowed for the direct identification of these phases through its ratiometric intensity signal [74]. The ratiometric response of F2N12S is particularly advantageous for imaging applications in both model and cellular membranes. Unlike intensity-based imaging, which can be affected by factors such as probe distribution heterogeneity, photobleaching, and variations in excitation light intensity, ratiometric imaging provides more reliable and consistent results.

***Molecular rotors*** are fluorophores exhibiting strong variations in their fluorescence quantum yields, spectra, and lifetime depending on their intramolecular rotation, which is a function of the environmental viscosity [103,104]. Since cell membrane viscosity affects the diffusion of protein, lipids, and other small biomolecules within the membranes and plays a crucial role in protein–protein interactions (e.g., via the formation of lipid rafts), monitoring it is essential. When incorporated into lipid membranes, these probes can effectively distinguish between disordered domains and more ordered regions. In more viscous media, the rotation of the molecular rotors is slowed, causing them to remain in the locally excited S1 state, which enhances the fluorescence quantum yield of the fluorophore within this phase. Typical examples of these probes are 9-(dicyanovinyl)-julolidine (DCVJ), its analogues like FCVJ, and some recently developed analogues of Laurdan and BODIPY.

An enhanced analogue of Laurdan, C-Laurdan-2, exhibits a specific affinity for lipid rafts in both live cells and tissues. As demonstrated in the study by Hwan Myung Kim and colleagues, this probe displays significantly brighter two-photon excited fluorescence in lipid rafts compared to non-raft membrane regions [75]. This brightness difference allows for high-resolution, direct visualization of lipid rafts within live cellular environments and in the CA1 region of hippocampal pyramidal neurons at depths ranging from 100 to 250 μm in live tissue. Furthermore, by substituting the carboxylate group of C-Laurdan-2 with a sulfonate, a new variant, S-Laurdan-2, was developed, which demonstrated higher photostability and slower internalization into the cytoplasm [75].

One major challenge with these fluorescent molecules is that plasma membrane imaging in living cells is often compromised by rapid endocytosis of the dyes. In this context, BODIPY dyes and their derivatives have gained significant attention due to their exceptional chemical and physical properties, including ease of functionalization, high molar extinction coefficients, tunable fluorescence quantum yields, visible-to-red excitation wavelengths, and remarkable photostability [104,105,106]. Kuimova’s group developed and thoroughly characterized a series of BODIPY-based molecular rotors that resist endocytosis thanks to a double positive charge on their hydrocarbon tail. Notably, they demonstrated the use of fluorescence lifetime imaging microscopy (FLIM) with the molecular rotor BODIPY-C10 in the plasma membranes of live *E. coli* bacteria to directly measure viscosity [78]. Despite its widespread use, BODIPY-C10 exhibits limited integration into liquid-ordered lipid phases and poor water solubility. To address these limitations, Polita et al. introduced a novel BODIPY-based probe, BODIPY-PM [79]. This probe features a long hydrophobic chain (C_12_H_25_) paired with a polar sulfonate group (SO_3_^−^). The combination of the charged sulfonate group, the hydrophobic tail, and the non-polar BODIPY core closely mimics the structure of natural phospholipids, ensuring stable incorporation and precise positioning of BODIPY-PM within the lipid bilayer.

The other class of molecular rotors mentioned above is represented by julolidine-based molecular rotors [107,108] like CCVJ [9-(2-carboxy-2-cyanovinyl)julolidine] and DCVJ [9-(dicyanovinyl)-julolidine]; however, their fluorescence intensity is influenced not only by viscosity but also by factors such as solvent dynamics and molecular interactions [80,107]. The other class of molecular rotors mentioned above is represented by julolidine-based molecular rotors [107,108] like CCVJ [9-(2-carboxy-2-cyanovinyl)julolidine] and DCVJ [9-(dicyanovinyl)-julolidine]; however, their fluorescence intensity is influenced not only by viscosity but also by factors such as solvent dynamics and molecular interactions [80,107].

## 4. Fluorescence-Based Techniques for Studying Lipid Rafts

Whether the coexistence of two liquid phases in model systems is immediately evident remained unclear in real systems for a long time. Initially, the presence of the raft-like domains coexisting with liquid-disordered regions was confirmed by microscopic observation and characterization in both free and supported lipid bilayers [109,110]. Through the ***widefield fluorescence technique***—in which the sample is entirely illuminated with light—Samsonov et al. [110] observed circular domains that were 2.5 μm and 4 μm in diameter in lipid bilayers containing rhodamine-phosphatidylehanolamine as a fluorescent probe.

The main limitations of conventional widefield microscopy concern the out-of-focus blurring; specifically, light from out-of-focus planes contributes to background noise, reducing image contrast [111]. Moreover, it lacks the inherent ability to optically section thick samples [112].

**Figure 4:**
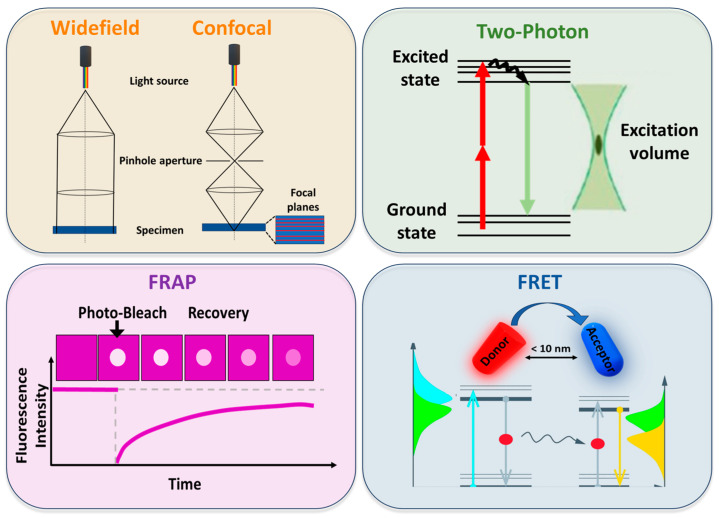
Schematic representation of the principles of widefield, confocal, and two-photon microscopies, together with fluorescence recovery after photobleaching (FRAP) and Förster resonance energy transfer (FRET) techniques.

***Confocal microscopy*** evolved as an alternative to widefield microscopy. As illustrated in Figure 4, confocal illumination occurs only at a resolution-limited point, which can be sequentially scanned in three dimensions throughout the sample [46]. Due to its ability, confocal microscopy is considered the most widely applied imaging technique because it allows live cell imaging with high spatial and temporal resolution, as well as optical sectioning and 3D reconstruction of images. The spatial resolution of confocal microscopy is fundamentally limited by diffraction, with the pinhole aperture playing a crucial role in determining the achievable contrast and optical sectioning. Under optimal conditions, axial resolution can reach approximately 0.8 μm, while lateral resolution can approach 0.3 μm [113].

While reducing the pinhole size improves the contrast, it also reduces the amount of light reaching the detector, potentially compromising image quality due to lower signal-to-noise ratios [114]. Additionally, confocal microscopy is inherently limited by a point-by-point acquisition method, leading to slow image acquisition times [115].

Confocal microscopy has been particularly useful for supported membranes or giant unilamellar vesicles (GUVs) [116]. Korlach et al. [116] were able to visualize micrometre-sized domains in lipid mixtures and to identify phases as ordered or fluid based on the known partition behaviour of appropriate fluorescent probes.

The lack of direct observation of micro-domains in living cells was explained by assuming that in real systems, raft-like domain size ranges in the nano-metre scale due to factors that in model systems are absent, such as the compositional complexity [117,118]. However, it was observed that under special physiological and compositional conditions [109,119] liquid-ordered domains are optically visualizable in live cells, as well. Indeed, confocal microscopy enabled the observation of microscopic domains both in model and living cells [119,120,121]. In particular, Hao et al. [119] were able to reveal the presence of domains in the plasma membranes of several mammalian cell types by means of lipid analogues and lipid-anchored proteins with varying fluidity preferences. Different methods have been developed based on the scanning system of confocal microscopy.

***Two-photon microscopy***, as shown in Figure 4, uses simultaneous absorption of two longer wavelength photons to excite a fluorophore [46]. It has become a powerful technique for imaging biological structures with greater depth (up to 1 mm [122] than confocal microscopy affords (approximately 100–200 μm [113]). An advantage of two-photon microscopy is the excitation of fluorophores through the use of two photons in the infrared range by avoiding sample photodamage. Moreover, infrared light has the ability to penetrate the sample to greater depths. Two-photon microscopy is ideal for exciting lipid probes like Laurdan [49,51] and C-Laurdan [70], which are typically activated by blue or near-UV light. This approach was used to detect the membrane domains in model membranes [123,124] as well as in living cells [94,125]. Furthermore, two-photon microscopy was exploited to investigate the order of different membrane systems [126] and to test new probes to visualize the membrane order [74,75].

Although it has significant advantages, two-photon microscopy has certain drawbacks. A practical limitation is the significant cost of appropriate ultrafast laser sources. Moreover, although two-photon excitation limits photobleaching to the focal plane, high-order absorption processes can produce accelerated photobleaching, especially in thinner samples [127]. This can result in increased photodamage compared to one-photon excitation [128], making the technique less advantageous for imaging thin specimens.

Another factor to take into consideration is saturation. It occurs when the excitation intensity is so high that all fluorophores within the focal volume are excited. The consequences are the loss of spatial resolution and the alteration of quantitative measurements [129].

***Fluorescence recovery after photobleaching (FRAP)*** exploits the photobleaching property of fluorophores. Following the bleaching of a specific region of interest, a subsequent fluorescence recovery is observed, caused by diffusion, interactions, or reactions of the surrounding fluorophores [130]. A schematic representation is reported in Figure 4. 

FRAP has been extensively employed to study protein and lipid diffusion mobility (diffusion coefficient of membrane lipids ~10^−7^–10^−8^ cm^2^/s and of membrane proteins ~10^−10^–10^−11^ cm^2^/s [131]), as well as to elucidate the domain structure of the plasma membrane. In the context of lipid rafts, FRAP has been applied to distinguish between the diffusion of raft and non-raft markers, as well as to characterize the factors, like cholesterol, which can influence membrane organization.

FRAP analysis gave insight into the nature of the interaction of Ras proteins with specific membrane anchorage domains. Niv et al. [132] employed studies on the lateral mobility of a constitutively active Ras isoform to clarify whether Ras anchoring associates preferentially with glycosphingolipid/cholesterol-enriched domains and concluded that only inactive H-ras interacts with lipid rafts; conversely, Roy et al. [133] concluded that the N-Ras isoform normally signals from lipid rafts and identified cholesterol as the interaction promoter with membrane domains.

Due to the challenge of directly studying lipid rafts, researchers have sometimes adopted a reversed experimental strategy by tracking raft-associated proteins. For instance, Kenworthy et al. [134] used FRAP to investigate the dynamic properties of lipid rafts by measuring the cell surface diffusion of putative raft-associated proteins tagged with green fluorescent protein (GFP) and Meder et al. [135] studied the long-range translational diffusion of several proteins and showed the domain coexistence in the apical membrane of epithelial cells.

Among the limitations of the FRAP technique is that the use of high-intensity lasers can damage samples and cause irreversible photobleaching of fluorophores, preventing their contribution to fluorescence recovery. Furthermore, if FRAP analysis assumes free molecular diffusion, it often does not reflect the real molecular movement, which is influenced by factors like interaction with other biostructures, leading to a non-ideal behaviour [136].

**Förster Resonance Energy Transfer (FRET)** is a photophysical phenomenon that monitors molecular dynamics and interactions down to a single-molecule level. The mechanism is based on the non-radiative energy transfer between the excited state of the donor fluorophore and an acceptor fluorophore via dipole–dipole interactions [137] (Figure 4). The energy transfer not only depends on the distance between donor and acceptor, but also on the spectral properties of the dyes and the relative orientation of their transition dipole moments [46]. The energy transfer event can result in different outcomes, like quenching of donor fluorescence, sensitized emission of the acceptor, reduction in donor lifetime, increase in donor fluorescence emission anisotropy, and depolarization of sensitized acceptor emission. Therefore, the design of a FRET experiment depends on which specific outcome is being monitored [137]. The application of FRET has significantly advanced our understanding of intermolecular interactions at scales smaller than 10 nm, approaching a single molecule scale. Indeed, this technique provided insights into the membrane heterogeneities in model and real systems [138]. Over the years, FRET has been applied not only to investigate the existence of domains but also to measure their size [139]. By analysing simple lipid models of the mammalian plasma membranes, Heberle et al. revealed that even the smallest difference in lipid composition can significantly alter phase coexistence and domain size. In addition to providing strong evidence for lateral lipid heterogeneity, FRET measurements demonstrated that the Lo/Ld segregation increases or decreases upon perturbation by varying cholesterol content or other agents like short-chain ceramides [140].

Beyond the great advantages, the FRET technique suffers from some limitations. For example, changes in environmental parameters (pH, ionic strength, temperature) can alter the fluorophore properties, leading to inaccurate FRET measurements [141]. Additionally, the high spectral overlap of donor and acceptor fluorophores generates a significant level of background noise that interferes with the FRET signal. Another limitation is the difficulty in taking into account all the factors in a FRET experiment, like the direct acceptor excitation, donor bleed-through, and the unknown relative orientation of donor–acceptor molecules, which can affect the accuracy of FRET measurements.

Although FRET has been fundamental in lipid raft research, the emergence of novel techniques like graphene-induced energy transfer (GIET) has opened new avenues for exploring lipid raft dynamics with unprecedented precision [142].

GIET represents a promising and powerful recently developed fluorescence–spectroscopic technique for investigating lipid rafts by leveraging the unique properties of graphene to study membrane behaviour at the nanoscale.

This technique is based on the electromagnetic nearfield coupling of a fluorescent emitter to excitons in a graphene sheet, acting as a dark quencher. Due to this coupling, the excited state lifetime of the fluorescent dye is greatly affected by the distance from the graphene layers. Full quenching occurs on the surface of the graphene. Thus, the precise localization of a fluorophore can be evaluated by measuring the lifetime of the dye and converting it in distance from the graphene. GIET can be used to track raft formation and dissociation in real-time. By tagging raft-associated lipids or proteins with fluorescent labels and using graphene as an energy acceptor, changes in energy transfer efficiency can be monitored to infer the size and composition of lipid rafts with sub-nanometre resolution [143].

Additionally, graphene energy transfer efficiency can reveal information about the fluidity of the lipid raft and how the raft’s composition (lipid types, cholesterol, or protein content) affects its behaviour under various conditions [144,145]. As the technique advances, it has the potential to uncover new insights into cellular signalling, drug development, and disease mechanisms related to lipid raft dysregulation.

## 5. Advanced Fluorescence Imaging Methods

### 5.1. Nanoscale Imaging of Lipid Rafts

Lipid rafts below 200 nm in size are too small to be visualized with traditional fluorescence microscopy. Techniques such as confocal and epifluorescence widefield microscopy are indeed restricted by the diffraction limit, a boundary in resolution established by Ernst Abbe in the late 19th century that limits visualization to structures larger than approximately 200 nm [146]. In recent decades, however, super-resolution fluorescence microscopy has overcome this barrier, enabling the observation of nanoscale structures. Key super-resolution techniques, including Stimulated Emission Depletion (STED) microscopy, Structured Illumination Microscopy (SIM), and Single Molecule Localization Microscopy (SMLM), offer unique strengths in spatial and temporal resolution, paving the way to explore different facets of membrane raft organization and dynamics in unprecedented detail [147,148]. A summary of the main characteristics of these super-resolution techniques is provided in Table 2.

**Figure 5:**
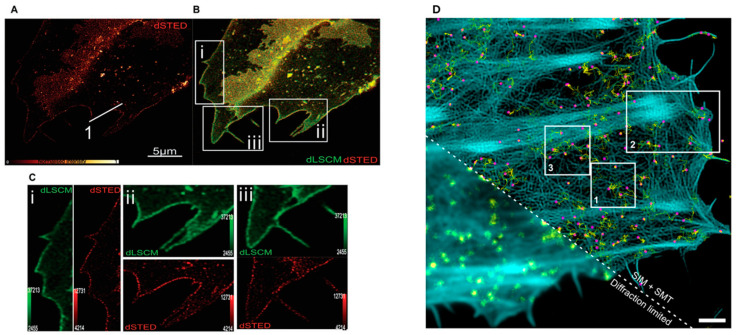
(**A**) Super-resolution 2D STED microscopy of cholesterol-rich nanodomains in the plasma membranes of living SH-SY5Y cells. (**B**) Super-imposed images of living cells visualized by both conventional deconvolved confocal (LSCM) and STED nanoscopy. (**C**) Magnified square insets (**i**–**iii**) in (**B**). Solid white line 1 in (**A**) has been used to show STED resolution improvement with nanodomains in the original paper. Adapted from Ref. [151] 2021 Copyright’s Wiley. (**D**) Single-molecule imaging of mXFP-TpoR diffusion (yellow) and the cortical actin cytoskeleton (cyan) of a representative cell analysed in Winkelmann et al. [161]. Magenta dots: single localization; yellow lines: trajectories faded in time. Scale bar: 2 µm. Adapted from Ref. [161] 2024 Copyright’s Nature.

STED microscopy is one of the pioneering super-resolution methods and operates by using two laser beams: a primary beam to excite fluorescence and a secondary, donut-shaped beam to quench fluorescence at the periphery of the excitation area [149]. This selective suppression allows only a narrow central region of fluorophores to emit detectable light, improving spatial resolution to a few tens of nanometres [150]. Using this approach, Lorizate et al. directly visualized cholesterol-enriched nanodomains in living cells (Figure 5A), providing compelling evidence of the existence of lipid rafts in physiological environments [151]. Furthermore, STED has been instrumental in observing molecular clusters thought to represent membrane rafts [152]. STED microscopy has indeed enabled high-resolution visualization of protein clusters potentially organized by membrane rafts, providing an indirect but powerful approach for probing the spatial organization and dynamics of membrane raft-associated structures. By combining STED with innovative click-chemistry methods, Saka et al. examined the spatial organization of membrane proteins, revealing the presence of stable, cholesterol-dependent multi-protein assemblies in the plasma membrane. These assemblies, anchored by the actin cytoskeleton, restrict protein diffusion and exhibit distinct localization patterns, with specific proteins preferentially occupying various regions within the assemblies. This work suggests that such mesoscale assemblies may represent a fundamental structural feature of the membrane, broadly influencing protein distribution and function. It is important to note that the STED technique relies on high-intensity laser light, which may induce thermal interference with the sample.

Another powerful super-resolution approach that can be used to visualize lipid rafts is provided by Structured Illumination Microscopy (SIM), which relies on interference patterns generated by sinusoidal illumination grids projected onto the sample [153]. By systematically shifting these patterned grids, SIM can extract high-resolution details from low-resolution images, effectively doubling the lateral resolution compared to confocal microscopy. Using SIM, Brown et al. observed the spatial distribution of the cortical actin meshwork surrounding natural killer TCR clusters, revealing active remodelling of membrane-proximal actin in these cells to facilitate lytic granule secretion [154]. Although SIM lateral resolution of around 100 nm is slightly lower than that of STED and requires some computational analysis effort, its relatively gentle illumination reduces photobleaching and phototoxicity, making it suitable for long-term imaging of live cells [155].

In parallel to STED and SIM, Single Molecule Localization Microscopy (SMLM) represents a family of techniques that localize individual fluorophores with high precision to construct super-resolved images [156]. Techniques like Stochastic Optical Reconstruction Microscopy (STORM) [157] and Photoactivated Localization Microscopy (PALM) [158] are prominent examples of SMLM. These methods work by randomly switching fluorophores on and off over successive image frames, isolating subsets of fluorescent molecules in each cycle. By calculating the centre position of each fluorophore, these positions can be combined to form a super-resolved image with resolutions as fine as 20 nm. SMLM has enabled the study of nanometre-scale membrane structures, such as protein clusters associated with rafts, revealing details about the spatial organization of membrane components. Sengupta et al. combined PALM with pair-correlation analysis to enable quantitative investigation of protein organization in the plasma membrane, allowing for the measurement of protein clustering, size, density, and distribution without needing absolute protein counts. The results of this study revealed distinct nanoscale organization of membrane proteins in COS-7 cells and showed significant changes in GPI-anchored protein arrangement under different conditions [159]. Using SMLM, Owen et al. quantified protein distribution and heterogeneity at the plasma membrane, revealing that tyrosine protein kinase clusters into sub-100 nm regions covering about 7% of the cell surface, demonstrating that SMLM methods allow mapping of nanoscale clustering [160]. Recently, Winkelmann et al. used SMLM with SIM to establish robust detection of receptor stoichiometries and probe fast dynamics at the plasma membrane (Figure 5B) [161].

Another variation of SMLM, Points Accumulation for Imaging in Nanoscale Topography (PAINT) [162], employs fluorescent probes that only emit signals when bound to specific targets. This can allow precise localization of membrane-associated molecules, further enriching the understanding of raft composition and dynamics. By applying PAINT, Winckler et al. analysed epidermal growth factor receptors (EGFRs), revealing their specific membrane localization and the formation of EGFR dimers upon epidermal growth factor (EGF) binding [163]. However, it is important to note that for the SMLM data to be valid, a sufficient staining density must be ensured.

Among the latest innovations in super-resolution microscopy, MINimal emission FLUXes (MINFLUX) stand out for its exceptional spatial and temporal resolution, with localization precision down to 2 nm [164]. MINFLUX combines the stochastic switching capabilities of SMLM with a doughnut-shaped excitation beam, which scans a small area around the target molecule. By positioning the doughnut’s dark centre near the fluorophore, it is possible to determine the fluorophore’s location with remarkable resolution, as fluorescence emission diminishes when the beam’s centre aligns with the target molecule. This technique can provide groundbreaking insights, such as the direct tracking of individual lipid molecules, helping to unravel the intricate dynamics of membrane rafts in living cells [165]. Despite these advantages, MINFLUX’s long acquisition times often require sample fixation, limiting its application in capturing the real-time dynamics of highly transient structures like membrane rafts.

**Table 2:** Super-resolution imaging techniques.

	STED	SIM	SMLM
Lateral resolution	≈20–30 nm	≈60–100 nm	≈20–30 nm
Microscope system	Scanning	Widefield	Widefield
Acquisition time	Seconds–minutes	Seconds–minutes	Minutes–hours
Dyes	Suited for emission stimulation	Conventional	Photoswitchable–Photoactivable
Post-processing	no	yes	yes
Limitations	High energy lasers	High computational analysis	Uniform labelling density
Refs	[149,166]	[153]	[158]

### 5.2. Probing Dynamics of Lipid Rafts

While spatial resolution is a key point, understanding the dynamics of lipid rafts also requires high temporal resolution. Techniques like Single Particle Tracking (SPT) [167] and Fluorescence Correlation Spectroscopy (FCS) [168] have significantly advanced our ability to probe the temporal aspects of membrane organization [169].

SPT involves labelling individual molecules, often with fluorescent tags, and monitoring their movement trajectories over time. This approach enables high spatio-temporal resolution insight into dynamic behaviours at the molecular level, such as diffusion, confinement, and interactions within cellular environments like membranes [170,171]. Studies using SPT have revealed that membrane proteins exhibit heterogeneous diffusion patterns due to obstacles or interactions with cytoskeletal structures [172].

A widespread alternative method is provided by FCS, a powerful technique that analyses fluctuations in fluorescence intensity within a small detection volume to measure diffusion coefficients and binding kinetics of fluorescently labelled molecules. This approach has been pivotal in quantifying the diffusion dynamics of lipid raft constituents [173,174]. Raster Image Correlation Spectroscopy (RICS) further builds on this by correlating intensity fluctuations in both time and space, allowing us to measure diffusion rates across a larger region [175,176].

These methods can be complemented with Fluorescence Lifetime Imaging Microscopy (FLIM) that provides additional layers of information by measuring the fluorescence lifetime of molecules, which can be influenced by factors such as molecular interactions and the local environment [177]. This technique has been utilized to observe lipid–protein interactions and phase separation in membrane models. When combined with super-resolution, SPT and correlation methods enable the study of raft dynamics over time, revealing how lipids and proteins move within and between these domains. An example of the synergy between super-resolution and correlation methods is STED-FCS, which uses STED microscopy to enhance spatial resolution in FCS measurements. For example, Eggeling et al. [166] applied FCS to investigate the diffusion rates of lipids within raft and non-raft domains, demonstrating that raft lipids exhibit significantly slower diffusion compared to their non-raft counterparts [166]. This finding supports the notion that lipid rafts serve as stable platforms for signalling molecules, enhancing the comprehension of their functional roles in cellular processes. By reducing the size of the excitation spot, STED-FCS achieves more precise measurements of molecular diffusion within membrane rafts. Muller et al. compared STED-FCS data on various fluorescent lipid analogues in the plasma membranes of living mammalian cells, revealing transient molecular complex formation. The strongest interactions were observed with sphingolipid analogues, showing cholesterol- and cytoskeleton-dependent binding, while other lipids, like gangliosides and galactosylceramide, exhibited weaker, less cholesterol- and cytoskeleton-dependent interactions, suggesting lipid-specific transient hydrogen bonding with membrane proteins [178].

In addition to these imaging approaches, advancements in probe design are continually expanding the range of molecules that can be targeted and visualized in live cells [179]. Fluorescent proteins and synthetic dyes with properties optimized for specific super-resolution techniques now allow for multicolour imaging, enabling the observation of interactions between different membrane components within the same sample. For example, dual-colour STORM or PALM can allow for simultaneous visualization of lipid and protein distribution, offering insights into how rafts organize specific signalling pathways. Although the goal of directly visualizing membrane rafts with high spatiotemporal resolution in live cells remains challenging, the continuous refinement of super-resolution techniques and probes is making it increasingly feasible. With further developments, it will soon be possible to achieve real-time, multicolour imaging of raft dynamics in live, unperturbed cells. This will not only advance our understanding of lipid raft biology but could also open new therapeutic avenues by providing insights into how these structures influence cellular signalling and disease progression.

## 6. Lipid Rafts: Recent Insights into Signalling, Diseases, and Infections Using Fluorescence Techniques

The following is a concise overview of the key scientific discoveries from the past decade in which the use of fluorescence approaches combined with other techniques has significantly advanced our understanding of lipid rafts, with particular emphasis on their implications in cases of major medical and scientific relevance.

### 6.1. Involvement of Lipid Rafts in Diseases

In recent years, lipid rafts have drawn growing attention due to their involvement in various processes, such as viral infections [180,181], Alzheimer’s (AD), and Parkinson’s (PD) diseases [182,183,184,185]. FRET, FLIM, and their derivatives have been effectively used to gain insights into the role of lipid rafts and their organization in several relevant biological processes, including the structural dynamics of receptors, the specific proteins or peptides related to neurological diseases, the pathogenesis in early AD and PD.

#### 6.1.1. Neuronal Diseases

As already described in Section 1.3, neurodegenerative diseases are frequently linked to changes in lipid composition that result in anomalous lipid raft organization and deregulation of signalling dependent on lipid rafts [186]. By using FRET-based protein–protein interaction analysis, Badawy et al. developed a new tool for detecting raft localization of S1P1R1 and showed that extracellular alpha-synuclein (α-Syn) causes expulsion of the S1PR1 receptor from the lipid raft domains [187]. α-Syn is a neuronal protein that is closely associated with the aetiology of Parkinson’s disease. To date, the normal function of α-Syn and the precise mechanisms through which it causes toxicity and cell death remain unclear. S1PR1 is a G-protein-coupled receptor activated by S1P, a sphingolipid that enhances cellular survival and highly regulates immune responses in the brain [187]. S1PR1 is partially localized to cholesterol-rich lipid rafts. Its expulsion from lipid rafts enables S1P stimulation and induces α-Syn binding to membrane-surface gangliosides and cholesterol imbalance in raft domains, resulting in S1PR1 uncoupling from G-protein. These findings are significant in shedding light on the role of extracellular α-Syn in the pathogenesis of synucleinopathies.

Fluorescence lifetime imaging microscopy (FLIM) combined with Förster resonance energy transfer (FRET) using two fluorescent probes targeted to liquid-ordered and -disordered domains of the plasma membrane revealed the specific localization of neuronal potassium KCN channels in lipid rafts [188]. This localization was shown to be regulated by the formation of a signalling complex with BACE1 [189], one of the major proteases that cleave the amyloid precursor protein (APP), leading to the accumulation of amyloid β (Aβ) fragments. Furthermore, the channel rearrangement and opening are regulated by changes in the order and thickness of the lipid bilayer. Ordered raft lipid domains are a preferred and functional location for potassium channels. When the order in lipid rafts is disrupted, HCN channels are localized in cholesterol-poor, disordered, non-raft lipid domains, leading to altered membrane functionality and contributing to disease progression [188].

The accumulation of amyloid-beta (Aβ) peptide, a hallmark of Alzheimer’s disease (AD), is thought to play a critical role in the onset and progression of the disease. Aβ is hypothesized to disrupt neuronal function by altering membrane fluidity, potentially leading to synaptic dysfunction. However, the precise effects of Aβ on the membrane remain unclear despite extensive studies using various model systems [190,191,192]. Ng et al. performed fluorescence correlation spectroscopy measurements to investigate this issue [193]. Although the concentrations of Aβ were sufficient to cause acute toxicity to the cells, the fluidity of both disordered and ordered phases of the plasma membrane was not altered. They concluded that alterations in membrane fluidity do not primarily mediate Aβ toxicity.

In addition to its well-known role in extracellular plaque formation, intracellular accumulation of Aβ has been implicated in the neurodegenerative processes underlying AD [194]. The internalization of extracellular Aβ through endocytosis is considered one of the possible mechanisms leading to the formation of intracellular Aβ deposits.

Recently, Nazere et al. found out that lipid rafts are involved in the cellular internalization of oAb42 by observing uptake reduction when lipid rafts are disrupted [195]. Thus, their study indicated the critical role of the lipid rafts in the process of oAb42 cellular uptake.

Although some studies suggest lipid rafts’ involvement in the formation of toxic amyloid aggregates or fibrils based on interaction with some specific lipids, including gangliosides [196], recent experiments have demonstrated the preferential interaction of amyloid-beta peptides in the liquid-disordered non-raft phase [197]. Furthemore, fluorimetric measurements by Staneva et al. [197] have shown the influence of Aβ peptides on the packing of the liquid-disordered phase depending on the presence of GM1.

#### 6.1.2. Viruses and Bacteria

As reported in the introduction, in addition to high levels of cholesterol and glycophospholipids, lipid rafts also contain a variety of cellular receptors that are crucial in various cellular processes, including immune response, inflammation, and specific signalling pathways [198]. Indeed, a growing number of viral and bacterial pathogens have been shown to enter host cells through clustered lipid rafts. In response to pathogens causing diseases, activation of different signalling pathways associated with the inflammatory response occurs, and the immune system sends in antibodies to attack and destroy bacteria; thus, the immune system plays a crucial role in host resistance to bacterial infection.

In this respect, fluorescence microscopy techniques have proved to be a useful and very cost-effective procedure for investigating and clarifying the molecular and cellular mechanisms by which pathogens interact with lipid rafts. As highlighted in Section 1.3, the role of lipid rafts in viral infections is pivotal. Enveloped viruses, i.e., viruses such as SARS-CoV-2, having a lipid envelope surrounding their capsid and entering cells by fusion (endocytosis) of viral and cell membranes, can use rafts to enter or exit target cells [199,200,201]. Elucidating how this fusion occurs can pave the way for developing targeted drug therapy strategies to prevent infection.

Recent insights into the endocytic interactions of viruses such as SARS-CoV-2 were provided by dSTORM imaging [202]. It was shown that cholesterol regulates viral entry through the localisation of the viral receptor (ACE2) in nanoscopic clusters of lipid rafts containing gangliosides (GM1). ACE2 moves to GM1 clusters when cholesterol levels are high, which is an ideal location for viral binding and endocytosis. Conversely, low cholesterol levels cause ACE2 to travel away from GM1 clusters, which is an unfavourable position for viral infection. Other authors, combining conventional microscopy methods (confocal, TIRF) with super-resolution microscopy techniques (ISM, STED, dSTORM), discovered that the virus employs clathrin-mediated endocytosis in the late entry pathway. Additionally, they observed that the viral receptor ACE2 is not localized in lipid rafts but rather in other disordered regions of the plasma membrane [203].

Among infectious diseases, drug-resistant infections are estimated to cause thousands of deaths every year. For instance, tuberculosis (TB), which is caused by Mycobacterium tuberculosis (Mtb), represents an enormous public health challenge due to the ability of Mtb to escape the host immune system and remain undetected in the lungs for decades, using cholesterol from the host as nutrient during infection [204]. Mtb cholesterol metabolism is important for maintaining persistent Mtb infection, but there is little knowledge about whether and how Mtb cholesterol metabolism can affect host immune responses. The time-resolved FRET technique was recently used to reveal the cholesterol metabolic MTB enzyme and the host nuclear receptor that are involved in inhibiting the host immune system and to clarify the mechanism through which they reduce cholesterol accumulation in lipid rafts, favouring their disruption and coalescence and suppressing the inflammatory response signalling pathways [205].

Methicillin-resistant Staphylococcus aureus (MRSA) is a human pathogenic micro-organism causing a variety of infections, such as skin and soft tissue infections, endocarditis, osteomyelitis, bacteraemia, and lethal pneumonia [206]. Due to its ability to internalize into lung epithelial cells, MRSA can avoid the effects of circulating antibiotics [207]. Understanding how to interfere with the bacterium’s entry would shed light on new therapeutic strategies. Through confocal microscopy, Goldmann et al. [208] demonstrated that the process of MRSA entry occurs thanks to the interaction between alpha-haemolysin (Hla)—a pore-forming toxin secreted by *S. aureus*—and Caveolin-1, which is one of the structural proteins within lipid rafts. Therefore, lipid rafts play a key role in the process of MRSA internalization into lung epithelial cells.

### 6.2. Lipid Rafts as Signalling Hubs

Lipid rafts act as dynamic platforms facilitating transmembrane signalling transduction because they harbour various receptors and regulatory molecules [209]. Improved understanding of lipid raft organization, their involvement in signalling processes, and their interactions with proteins and receptors will help in the development of new strategies for effective prevention and treatment of diseases. 

Bag et al. [210] investigated the role of lipids and proteins in transmembrane (TM) signalling in mast cells. TM signalling is stimulated by the phosphorylation of antigen-crosslinked immunoglobulin E receptor (IgE-FcεRI) complexes by lipid-anchored Lyn kinase. Thanks to imaging fluorescence correlation spectroscopy (ImFCS), the authors provided insights into the role of lipids in stimulated Lyn/ FcεRI coupling, confirming that the Ag crosslinking stabilizes liquid-ordered-like nanodomains around the clustered IgE-FcεRI. Moreover, they demonstrated that Lyn partitioning into Lo-like nanodomains near FcεRI clusters enhances cytosolic protein–protein interactions, facilitating efficient kinase–receptor coupling. Therefore, Lyn’s preferential partitioning into the Lo phase is essential for initiating stimulated TM signalling.

During brain development, Netrin-1, a guidance cue, interacts with UNC5 receptors to regulate axonal growth. Hernaiz-Llorens et al. [211] used the combination of confocal microscopy, FRAP, and single-particle tracking photoactivation localization microscopy (sptPALM) to characterize the membrane distribution of all UNC5 receptors. They found that the distribution of all UNC5 receptors into raft microdomains has functional consequences for axonal chemo repulsion against Netrin-1. They demonstrated that the organization of UNC5 into microdomains is functionally relevant for the development of cerebellar structures.

Wnt3 is another fundamental signalling molecule (morphogen) that plays a key role in various biological processes like cell proliferation, cell fate specification, and differentiation over embryonic induction to neural patterning [212]. The palmitoylation (post-translational lipid modification) of Wnt3 by Porcupine (a membrane-bound O-acyl-transferase) is essential for its intracellular membrane trafficking. Ng et al. [213] Wnt3 localization in the cholesterol-dependent domains in the apical membrane of the cerebellar cells in live transgenic zebrafish embryos by means of single-plane illumination microscopy–fluorescence correlation spectroscopy. Moreover, they found that Wnt3’s association with domains strongly depends on its palmitoylation; thus, they suggested that membrane localization of Wnt3 is crucial for its extracellular trafficking and subsequent signalling in neighbouring cells, ensuring proper neural development.

Soyasaponins (SSs) are a family of phytochemicals found extensively in soybeans, and their well-known anti-inflammatory activity [214] has been shown to be mostly associated with their regulation of the signalling cascade of Toll-like receptor 4 (TLR4) [215,216]. TLR4 generally localizes on the cell surface of macrophages, and palmitic acid, being its natural endogenous ligand, activates its signalling pathway. Lipid rafts are considered indispensable for the activation of TLR4, and lipid-rafts-mediated TLR4 signalling has become a promising therapeutic target.

Gu et al. [217] showed, through FRET and confocal microscopy, that SS-A1 can inhibit lipid raft recruitment and dimerization of TLR4 in the PA-stimulated inflammatory Raw264.7 macrophage cell line thanks to their influence on the formation, clustering, and size of lipid rafts by maintaining cholesterol homeostasis.

A key challenge currently being addressed by several research groups is understanding how specific phospholipids, such as long-chain polyunsaturated fatty acids and oxidized acyl chains of phospholipids [218] or alcohol [219,220], control the formation of lipid rafts and impact the dynamics of transient lipid rafts. These interactions, which have significant implications for inflammation, immunological pathways, and receptor-mediated processes [219], remain complex and not yet fully elucidated.

Ethanol has a profound impact on the function of various neurotransmitter and neuromodulator systems in the CNS. Ethanol has been proposed to affect surface receptor signalling by altering the lateral organization of proteins and lipids in the plasma membrane [221]; in particular, ethanol reduces cholesterol perturbation of the structure of lipid rafts.

Tobin et al. [222] demonstrated that effectively relevant concentrations of ethanol lead to a lateral reorganization of the plasma membrane and cytoskeletal proteins by means of Photoactivated Localization Microscopy with pair-correlation analysis (pcPALM). Thus, their study suggests that alterations in membrane lipid organization and actin cytoskeletal dynamics play a significant role in alcohol-induced cellular effects.

The milk-derived bovine lactoferrin (bLf) is an iron-binding glycoprotein with the property of being selectively cytotoxic to cancer cells [223]. The plasmalemmal V-ATPase (an ATP-driven proton pump localized intracellularly and at the plasma membrane in metastatic cancer cells) has been identified as the critical target of bLf.

Santos-Pereira et al. [224] observed the localization of V-ATPase on lipid rafts in highly metastable static cancer cell lines through confocal microscopy; moreover, their immunofluorescence studies demonstrated that intracellular trafficking is perturbed upon V-ATPase inhibition by bLf binding, leading to accumulation of cholesterol inside the endo/lysosomes, its subsequent depletion from the plasma membrane, and disruption of lipid rafts. These insights suggest that Lf-based therapies could be a promising strategy for targeting V-ATPase-positive cancer cells, offering a novel approach to cancer treatment.

### 6.3. Targeting Lipid Rafts Using FRET-Based Biosensors

FLIM and FRET and their derivative techniques are among the most promising optical techniques that neuroscientists can employ to investigate the structure and function of in vivo and in vitro complex biological systems. They are increasingly used in neurobiology to study protein–protein interactions or conformational changes in protein complexes and to keep track of FRET-based biosensors that have been genetically encoded.

FRET-based biosensors are being developed to detect specific biomolecules or to monitor changes in the cellular or molecular microenvironment.

These biosensors rely on donor and acceptor molecules, typically fluorescent proteins, fluorescent nanomaterials such as quantum dots (QDs), or engineered small molecules positioned in proximity [225].

Upon interaction with the target molecule, the distance between the donor and acceptor changes, altering FRET efficiency and the fluorescence intensity of the acceptor. Based on this change, it is possible to detect and quantify the target molecules. Using acylation and prenylation as lipid modification signals, FRET biosensors can be precisely localized inside or outside lipid rafts, providing a powerful tool for studying dynamic lipid raft signalling events in live cells with high spatiotemporal resolution [226].

To gain deeper insights into the complex mechanisms of platelet-derived growth factor receptor (PDGFR) activation and its involvement in intracellular signalling pathways, it is necessary to develop innovative tools that enable real-time dynamic monitoring of receptor activation and interactions.

Seong J. et al. developed a novel FRET-based molecular biosensor that includes a PDGFR autophosphorylation site. Their study demonstrated that significant PDGFR activation occurs both within and outside lipid rafts following PDGF stimulation [227]. Moreover, they found that the regulation of PDGFR activity is mediated by integrin-dependent signals and is restricted to non-raft lipid domains, suggesting that lipid rafts serve as specialized signalling platforms that help control cellular functions with precision. The activation of PDGFR is triggered by the binding of PDGF [227]. The membrane microdomains where PDGFRs reside and control intracellular signalling pathways is the subject of debate. PDGF is one of the major serum growth factors that promote cell proliferation, survival, and migration, particularly in cells of mesenchymal origin [228]. The dysfunction of PDGF signalling has been implicated in various pathological conditions, including cancer, fibrosis, neurological conditions, and atherosclerosis [229]. Ligand binding leads to autophosphorylation of the receptors on tyrosine residues, and this event induces activation of several signalling molecules.

Kim et al. provided another demonstration of how FRET-based sensors can effectively study the role of lipid rafts in regulating cellular functional activity [230]. Using a genetically encoded FRET sensor, they proved that the FAK protein (a crucial regulator of vital cellular processes) and Ca^2+^ signals are selectively activated in the lipid raft domains, while the non-raft domains show minimal activity. Their results suggest that both FAK and the rigidity and integrity of lipid rafts mediate in situ Ca^2+^ signalling activation during the cell adhesion process. Furthermore, they also proved the crucial role of lipid raft cholesterol in Ca^2+^ mobilization and control during the cell–matrix adhesion process.

## 7. Conclusions

Lipid rafts are pivotal microdomains in membrane biology, acting as dynamic platforms for crucial cellular processes and playing significant roles in both physiological and pathological conditions. Their involvement in diseases such as cancer, neurodegenerative disorders, and viral infections underscores their importance as a target for research and potential therapeutic interventions. The advent of advanced fluorescence-based techniques, particularly super-resolution microscopy, has revolutionized the study of lipid rafts by enabling unprecedented insights into their organization, dynamics, and interactions at the nanoscale.

A cornerstone of this progress lies in the development of innovative fluorescent probes tailored for lipid raft studies. These probes, with high specificity and sensitivity, have enabled researchers to visualize the intricate composition and behaviour of lipid rafts within the complex environment of living cells. Their non-invasive nature facilitates real-time observation under physiologically relevant conditions, providing invaluable quantitative data that were previously unattainable with traditional methods.

Super-resolution techniques, such as STED, SIM, PALM, and STORM, have further expanded the boundaries of what can be resolved in biological systems. By surpassing the diffraction limit of conventional microscopy, these methods enable the precise localization of raft-associated molecules and the mapping of their dynamic interactions within membranes. When combined with advanced fluorescent probes, these techniques enable a holistic understanding of lipid raft functionality, bridging the gap between molecular-scale details and cellular-level processes.

As advancements in fluorescent probe design and super-resolution imaging methodologies progress, they hold the potential to revolutionize our understanding of lipid rafts. These cutting-edge tools promise to reveal unprecedented details about the spatiotemporal dynamics, molecular composition, and functional roles of lipid rafts in cellular membranes.

Such breakthroughs will deepen our knowledge of membrane biology, shedding light on how lipid rafts orchestrate critical processes like signal transduction, protein trafficking, and pathogen–host interactions. Moreover, these insights are expected to catalyse the development of innovative therapeutic strategies targeting lipid-raft-associated mechanisms, particularly in diseases such as cancer, neurodegeneration, and infectious disorders.

By bridging molecular-level discoveries with clinical applications, the ongoing evolution of these technologies heralds a transformative era in both basic research and translational medicine, offering new avenues to manipulate lipid raft dynamics for therapeutic benefit.

## Figures and Tables

**Table 1:** Fluorescent membrane probes: phase partitioning Properties.

Name	Partitioning	Reference
***Probes for lipid components***		
Cholera toxin B (CT-B)	**-**	[52,53]
BODIPY-GM1	Lo	[54]
Filipin	**-**	[55,56,57]
Cholestatrienol (CTL)	Lo	[58,59]
Dehydroergosterol (DHE)	**-**	[58,59,60]
(NBD)-cholesterol	Ld	[61]
TopFluor-Cholesterol (TF-Chol)	Lo	[62]
BODIPY-SM	Lo	[63]
ATTO_488_-SM	Ld	[64]
ATTO_594_-SM	Ld	[64]
***Probes Selective to Lo Phase***		
Terrylene	Lo	[65]
Naphthopyrene (NAP)	Lo	[65]
***Solvatochromic probes***		
Laurdan	Lo/Ld	[66,67]
Prodan	Lo/Ld	[68,69]
C-Laurdan	Lo/Ld	[70]
di-4-ANEPPDHQ	Lo/Ld	[71]
Nile Red	Lo/Ld	[72]
NR12S	Lo/Ld	[73]
F2N12S	Lo/Ld	[74]
***Molecular rotors***		
C-Laurdan-2	Lo/Ld	[75]
S-Laurdan-2	Lo/Ld	[76]
FCVJ	**-**	[77]
BODIPY C10	Ld	[78]
BODIPY-PM	Lo/Ld	[79]
CCVJ	**-**	[80]

## Data Availability

Not applicable.
